# The impact of public involvement in health research: what are we measuring? Why are we measuring it? Should we stop measuring it?

**DOI:** 10.1186/s40900-020-00239-w

**Published:** 2020-10-27

**Authors:** Jill Russell, Nina Fudge, Trish Greenhalgh

**Affiliations:** 1 Institute of Population Health Sciences, Barts and The London School of Medicine and Dentistry, Queen Mary University of London, London, UK; 2 Primary Care Health Sciences, University of Oxford, Oxford, UK

**Keywords:** Impact, Patient and public involvement, Public involvement

## Abstract

As public involvement in the design, conduct and dissemination of health research has become an expected norm and firmly enshrined in policy, interest in measuring its impact has also grown. Despite a drive to assess the impact of public involvement, and a growing body of studies attempting to do just this, a number of questions have been largely ignored.

This commentary addresses these omissions: What is the impact of all this focus on measuring impact? How is the language of impact shaping the debate about, and the practice of, public involvement in health research? And how have shifting conceptualisations of public involvement in health research shaped, and been shaped by, the way we think about and measure impact? We argue that the focus on impact risks distorting how public involvement in health research is conceptualised and practised, blinding us to possible negative impacts.

We call for a critical research agenda for public involvement that [a] considers public involvement not as an instrumental intervention but a social practice of dialogue and learning between researchers and the public; [b] explores how power relations play out in the context of public involvement in health research, what empowerment means and whose interests are served by it, and [c] asks questions about possible harms as well as benefits of public involvement, and whether the language of impact is helpful or not.

## Plain English summary

The involvement of patients and the public in health research is common practice and a key requirement made by research grant funding bodies. As public involvement has grown, so too has a desire to measure and demonstrate its impact – this could be the impact involvement activities have on research processes and outcomes, the impact involvement has on participating members of the public and researchers, or the impact involvement has on addressing power imbalances between the researcher and the researched.

In this commentary, we look at what effect this focus on impact is having on rationales for doing public involvement in the first place and how public involvement is done. Measures of public involvement which are easy to count, such as numbers of people involved, tend to be favoured. However, this tells us little about how patients and the public have changed the course of research studies. Certain kinds of impact, such as how do public involvement activities change power relations and empower the public, are largely not being captured.

We call for a critical research agenda for public involvement – one that acknowledges:

- different rationales for public involvement, and how the desire to measure effects might be shaping these rationales.
- that there might be negative impacts of public involvement as these are largely unreported.
- the impact of power and in particular how power relations play out in research settings.

## Background

As public involvement in the design, conduct and dissemination of health research has become an expected norm and firmly enshrined in policy in the UK [1, 2] and internationally [3], interest in measuring its impact has also grown. Advocates of public involvement in health research are keen to see its benefits demonstrated; sceptics anticipate having their doubts confirmed; and others would like to better understand whether and how public involvement impacts on research processes and outcomes, on those involved, and any broader societal effects [4, 5].

The international literature on how to evaluate the impact of public involvement has more than tripled in recent years [6]. The question has been addressed in systematic reviews [7–14], realist evaluations [15–17] and studies of stakeholder views [18, 19]. Some studies have developed practical guidance for researchers to incorporate assessment of the impact of public involvement in their research [20], and at least 65 frameworks (summarised in a recent systematic review [21]) have been developed for assessing the nature and impact of public involvement in health research.

Together these studies offer a substantial evidence base on certain aspects of the impact of public involvement in health research. They have identified, for example, how public involvement can increase recruitment to clinical trials [8, 22, 23], make research more relevant and appropriate for users [14, 24], help to formulate research questions and shape and reshape study design [25, 26], and provide insights to inform and develop analysis [27]. However, a significant proportion of the available evidence about impact is considered to be anecdotal and weak [28, 29], and there are frequent and widespread calls for more robust methods and instruments to capture and measure impact [30–32]. A recent BMJ editorial on the subject of impact of public involvement in health research concluded: *“if we are serious about involvement, we need to be equally serious about evaluation [of impact]”* [6].

Questions that have been largely ignored by the existing evidence base, and which provide the starting point for this paper, include the following: What are the consequences of all this focus on measuring impact? How is the language of impact shaping the debate about, and the practice of, public involvement in health research? And how have shifting conceptualisations of public involvement in health research shaped the way we think about and measure impact?

Critical academics, prompted by the inclusion of impact in the Research Excellence Framework (a system for assessing research quality in UK universities), have argued against measuring impact. Formal measurement of impact may distort practice and draws us into the mindset and practices of performativity. As Fielding put it, *“it valorises what is short-term, readily visible and easily measurable... it has difficulty comprehending and valuing what is complex and problematic”* [33]. Reports tend to emphasise positive impacts and to under-report or neglect what might be construed as negative [34]. Rarely considered – perhaps because they are hard to measure – are long-term but potentially far-reaching influences (positive and negative) on the culture of research itself. Impacts may get overblown in an attempt to secure further funding – a phenomenon referred to as ‘impact sensationalism’ [35]. An insistent focus on measuring impact can mean that the collection of other equally important evidence, such as data on implementation processes, is compromised [36].

In this commentary, we contend that there are comparable arguments to be made in relation to measuring the impact of public involvement in health research. The current emphasis on impact, we believe, risks distorting how public involvement in health research is conceptualised and practised, and blinds us to possible negative impacts of public involvement in health research. In making these arguments it is not our intention to deny the importance of exploring the effect of public involvement in health research, nor to undermine the value of work undertaken by researchers in this field. Rather, we wish to draw attention to the particular ways in which the debate about impact has been framed, and to highlight the associated risks.

The arguments we present have emerged from an historical analysis that two of us (JR and TG) undertook of the intended impact of the National Institute for Health Research (NIHR) public involvement policy, commissioned by NIHR to provide a baseline for further work on assessing impact of public involvement [37]. Our focus is therefore primarily, though not exclusively, oriented to NIHR policies and practices, widely cited as exemplars, both nationally and internationally, of public involvement in health research [3, 10, 16, 38]. We also draw on the second author’s ethnographic work on involving stroke survivors in service development and research [39]. Throughout our commentary we follow NIHR in adopting the term ‘public involvement’ to include patients, service users, survivors, carers and family members [1], while mindful of the distinctions that are drawn between the categories of ‘patient’ and ‘public’ [40]. The broad term of public involvement intersects with, but does not necessarily equate with, approaches such as patient-oriented research, integrated knowledge translation research, lay representation, citizen engagement, co-production and co-design.

## How public involvement in health research is conceptualised

The majority of studies of the impact of public involvement in health research have focused on its impact on the research process (for example, on recruitment and retention rates), and on the quality, validity, relevance and utility of research [7, 8]. Public involvement is conceptualised as a means to the end of achieving better research – or, to put it in philosophical terms, public involvement is seen through a ‘consequentialist’, or ‘benefits-based’ lens [41]. In line with the norms of evidence-based medicine, which is seen as the gold standard for measuring effectiveness in health research, public involvement is positioned as an intervention that has a potentially fixed and measurable effect size [28].

This conceptualisation of public involvement has appeal and persuasive power to researchers – it focuses on what is familiar to them in terms of scientific method, and what is likely to matter to them most: the question of whether public involvement leads to better research [8]. However, the risk is that other, equally important, conceptualisations of public involvement are obscured. Mathie et al. argue that: *“defining consumer involvement outcomes solely in terms of research quality ignores the rights of those being researched or likely to benefit from the research”* [10]*.*

An alternative ‘democratic’ or ‘rights-based’ framing of public involvement draws on philosophies of justice, human rights and empowerment, and sees public involvement not so much as a means to an end (better research) but as an *end in itself* [42]. From this perspective, public involvement is *“primarily concerned with people having more say in agencies, organisations, and institutions which impact upon them and being able to exert more control over their own lives”* [43]. This conceptualisation of public involvement necessarily shifts the attention of impact studies from the research endeavour onto patients and the public and the wider community. For example, studies have highlighted how involvement in health research may provide a life focus for patients and the public, can impact positively on self-confidence, and on knowledge about best evidence relating to treatment and care [14, 44, 45]. Furthermore, it can provide support and friendships, and facilitate the learning of new skills, such as communication, presentation, and research skills [18]. Whilst these sort of positive impacts on individual experience may be a first step towards the democratising of research processes, direct forms of participation do not necessarily address the democratic deficit at a collective level, and arguably might perpetuate inequalities if only certain people benefit from involvement. Studies have suggested that organisation-led involvement, practiced within the ‘norms of bureaucracy’, encourages a certain type of patient or citizen to participate, those who feel comfortable with the business meeting or committee format [46]. This means that those who get involved in health research are predominantly white, middle class, retired people, often from a health or research background, constituting an ‘unrepresentative minority’ [47, 48]. Citizens and community members who routinely go unheard – those with extremes of age, complex health/social care needs, limited English speakers, the homeless, those without full citizenship rights, those whose illness makes communication or understanding difficult, or with stigmatising illnesses - are further actively discouraged to participate [46].

Some argue that if involvement is conceptualised as a democratic right, with intrinsic value, and an end in itself, no further evaluation of impact is needed [41]. Others have suggested that it is inappropriate to consider public involvement in health research as an intervention with measurable impacts, questioning “*the appropriateness of applying scientific enquiry to a social, collaborative partnership, where mutual learning takes place during personal interactions”* [18]. In this vein, Staley and Barron suggest that we should instead conceptualise public involvement as conversations between researchers and the public that support two-way learning. The learning that takes place from such conversations, they suggest, can be captured through in-depth qualitative methods, but is always going to be difficult to quantify and measure. Staley and Barron consider public involvement in health research, and the learning to emerge from it, as evolutionary, unpredictable, subjective and context-specific. They argue that we need to move away from an instrumental conceptualisation of patients and the public as *‘the intervention’*, and from the view that experimental methods, and particularly randomised controlled trials, are an appropriate way of assessing impact. They conclude: *“trying to standardise involvement processes as ‘methods’ and to objectify the outcomes, may be akin to ‘forcing a square peg into a round hole’. The richness and value of subjective learning needs greater recognition”* [28]*.*

Friesen and colleagues make similar criticisms of the effect of the impact agenda on conceptualisations of public involvement [5]. They argue that the dominant focus on measuring the impact of public involvement is harmful because of the way in which it has diverted attention away from ethical reasons for public involvement, towards a narrow focus on epistemic justifications. Epistemic justifications for public involvement, which approximate to the benefits-based conceptualisation of public involvement discussed above, focus predominantly on the impact that involvement has on the research process and outputs, on what Friesen et al. refer to as the three Rs: rigour, relevance and reach. But, they argue:

“Service users have not fought for a voice at the table merely to help improve the research process, but because they have a right to be there. This suggests that what justifies involvement is much larger than that captured by the epistemic focus of those seeking to evaluate the impact of participatory research” [5].

Whilst Friesen and colleagues are talking specifically about participatory research in psychiatry, we suggest that their arguments are applicable to public involvement in health research more generally. As these authors point out, the *ethical* reasons for involvement are to do with democratic rights and empowerment. Paying serious attention to this conceptualisation of involvement, they argue, means looking beyond questions of impact, to broader questions about imbalances of power.

Changing power relations between researchers and the researched has long been a motivating aim of user social movements, particularly those of people with disabilities and mental health service users [43]. The aim has been for public involvement to progress up Arnstein’s ‘ladder of participation’, a conceptual framework for understanding involvement dating from the 1960s [49]. Low down on the ladder are activities such as informing and consultation, frequently perceived as tokenistic forms of public involvement, moving up to more meaningful and genuine forms of power-sharing such as partnership, delegated power and ultimately citizen control of the research agenda.

While some commentators argue that the past decade has seen real shifts towards the democratisation of health research [4], others are less confident that there has been any fundamental shift in power relations from the scientific community to the public. From her analysis of NIHR activity, Green for example argues that, despite an increasingly progressive rhetoric, as evidenced in the attention given to methods such as co-production and co-design, and despite a body of evidence demonstrating that it is increasingly the norm for the public voice to be incorporated into various stages of the research process, *“there has not, however, been a concomitant transformation of the social relations of research, as envisaged by the emancipatory research movement”* [42]. Boaz and colleagues’ interview study of researchers involved with NIHR Biomedical Research Centres concluded that despite *“changing currents on the surface”*, there has remained active resistance to sharing power and control in the process of knowledge generation [50]. Similarly, a recent study of the development of public involvement in a London based mental health biomedical research centre reported that:

*“PPI remained localised and under resourced and there was a reluctance to change working practices which resulted in perceptions of tokenism. Service users faced conflicting expectations and were expected to assimilate rather than challenge the organisation’s ‘biomedical agenda’”* [51]*.*

Notwithstanding these snippets of evidence, we still know very little about the impact of public involvement on power relations between researchers and the public, because this is rarely the focus of impact research. A reviewer of an earlier draft of this paper commented that whereas researchers and research funders have to a large extent accepted and come to value a certain kind of *patient* involvement (in the sense of contributing the lived experience of illness to the research agenda of a particular condition [52]), the place of *public* involvement (in the sense of accountability to critical and questioning voices from wider society) remains awkward.

Indeed, some studies have even suggested signs of movement away from the democratisation of health research. Our historical analysis of NIHR policy suggested that whereas earlier policy documents emphasised a rights-based discourse of involvement (epitomised by the phrase ‘nothing about us without us’), we detected a more recent concern with the duties and responsibilities of users and citizens [37]. For example, the strategic goal of NIHR policy identified in ‘Going the Extra Mile’ is an expectation that by 2025 everyone using health and social care *“to choose to contribute to research”* through a number of avenues. The public, as users of health services, are to be ‘empowered’ to ‘seize the opportunities’ to engage and become actively involved in research:

*"By 2025 we expect all people using health and social care, and increasing numbers of the public, to be aware of and choosing to contribute to research by:*

- Identifying future research priorities and research questions
- Informing the design and development of innovations
- Participating in research studies
- Advocating for the adoption and implementation of research in the NHS" [2].

It seems that public involvement policy here is ‘empowering’ people to become responsible ‘active citizens’ who contribute to the national research endeavour [53]. We can see a subtle shift in the conceptualisation of empowerment, away from any emancipatory understanding of the term, towards a restricted one to do with ‘participating in research studies’ and ‘informing’, activities that are more amenable to measurement. And as a consequence, questions about how public involvement might enable the sharing of power, who power is being shared with, and in what ways, are easily side-stepped [5].

## How public involvement in health research is practised

Approaches to measuring impact of public involvement in health research shape not only how we conceptualise public involvement but how it is practiced. The language of impact demands that effects can be formally measured. In other fields the arguments of how a preoccupation with measurement shapes/distorts practice have been well rehearsed – ‘teaching to the test’ in education [54], the ‘gaming’ of quality targets in health care [55], and so on [56–58].

In the field of public involvement in health research we see how, for example, in NIHR reporting on public involvement activity, the focus is invariably on quantifying impact – the number of training workshops and conferences held each year, the number of people employed as public involvement leads, facilitators and advisers, and quantifying the work of the NIHR Research Design Service [59–61]. What gets recorded is what can be measured (and therefore defined as impact) and the ‘backwash’ effect of this is that what gets done is what can be measured. Wilsdon and colleagues argue that in public policy the focus on *“the ‘hardware’ of engagement – the methods, the focus groups, the citizens’ juries that can give the public a voice in science policy and decision-making”* has too often been at the expense of *“the ‘software’ – the codes, values and norms that govern scientific practice, but which are far harder to access and change”* [62]. These “software” elements are also far harder to measure and demonstrate impact, and therefore tend to get neglected in favour of easier aspects of practice to measure and quantify. Chubb and Derrick’s analysis of research impact in higher education suggests that there may also be a gender dimension at play here, given the gendered associations with notions of ‘hard’ and ‘soft’:

*“gender may play a role in the prioritisation of ‘hard’ Impacts (and research) that can be counted, in contrast to ‘soft’ Impacts (and research) that are far less quantifiable, reminiscent of deeper entrenched views about the value of different ‘modes’ of research”* [63].

A recent development with public involvement in health research in the UK has been the setting of national standards by NIHR [1]. While the authors of these standards explicitly say that *“﻿They are not designed as rules, or to provide fixed ideas about public involvement in research”* [64], the danger is that they will do exactly that. The advantage of standards is that they offer concrete examples of *how* public involvement can be undertaken. But their disadvantage is that they can become prescriptive; they define and bound activity in a constraining way, institutionalising the rules of the game [65]. McCoy and colleagues go further in their criticism of the standards, arguing that they *“fail to address fundamental questions about when, why and with whom involvement should be undertaken in the first place”*. This lack of justificatory context, they argue, feeds the problem of tokenism in public involvement practice – it encourages researchers to undertake involvement activities without necessarily being clear about or reflecting fully on their purpose. The danger is that the standards promote *“an unreflective ‘more the merrier’ attitude in relation to involvement”* [66].

## The impact of public involvement in health research – what is not being captured?

Public involvement is typically presented as being unquestionably a good thing in relation to healthcare in general and health research in particular [67]. Consequently, impact tends to be unproblematically equated with benefit. This can be seen in the NIHR Public Involvement Impact Working Group’s definition of impact of public involvement in NIHR health and social care research: “*The changes, benefits and learning gained from the insights and experiences of patients, carers and the public when working in partnership with researchers and others involved in NIHR initiatives”* [68]*.* In this definition there is no mention of negative impacts. Interestingly, in one of the few and now somewhat old reviews of negative impacts (from 1997 to 2009), Staniszewska et al. found that more discussion of negative impact occurred in the earlier papers they reviewed. It is unclear, they suggest *“why a reduction has occurred at a time when interest in impact has expanded”* [69]. One explanation, we would suggest, is that as public involvement has become more formalised into institutional structures and research practices, it has perhaps become harder to speak critically of it. And while the NIHR national standards for public involvement at least acknowledge that *“we can learn from both positive and negative impacts”*, it is disappointing that there is no further discussion of possible negative impacts [1].

A few researchers have identified some of the negative impacts of public involvement on those who get involved: feelings of overwork and frustration at the limited opportunities to influence the direction of research; feelings of being marginalised, confusion and conflict due to lack of clarity about the lay role, the burden of responsibility and duty, as well as time and financial burdens [18, 44, 70]. Ashcroft and colleagues suggest that more needs to be done to mitigate the possible negative effects of involvement, by for example ensuring language is clear and not excluding, giving regular feedback, and ensuring people involved have sufficient time to digest information [45]. Concerns have also been expressed by both the public and researchers that public involvement can sometimes appear to amount to little more than a ‘tick box’ exercise, with researchers going through the motions of involvement to satisfy the reporting requirements of funding bodies and ethics committees, rather than meaningful and robust engagement with the processes of involvement [71].

Others have drawn attention to the possible negative impact of involvement on researchers themselves, in terms of the additional resources, time, and skills required, and the stress and tensions that can arise in negotiating the divergent interests involved. Moreover, it is suggested that public involvement challenges the ‘science’ of research and whose knowledge counts; Bekkum and Hilton for example found *“numerous and sometimes conflicting concerns about public knowledge deficits and their biases, emotions and personal interests potentially damaging the integrity of science”* [19]. Similarly, Oliver et al. suggest that coproduced research can be regarded by the academy as “partisan and biased” and thus of lower quality than “‘real’ or ‘pure’ research” [72].

Perhaps the most important criticism of public involvement in health research is that, rather than being empowering or emancipatory, it runs the danger of having precisely the opposite effect. Fudge’s research of stroke survivors’ involvement in service development and research suggests that the way in which the resources of service users are sought through public involvement mechanisms can ultimately inhibit their capacity for protest, and thus, she argues, has the overall effect of containing and quietening radical social movements [39, 73] Mechanisms such as time-control, allotted slots for patients to speak, and pre-set agendas served to ensure patient discussions were contained and did not go astray. The ritual structure of meetings that public involvement organisers employed (in terms of orientation, time and content), ensured that public involvement was directed towards researchers’ own productive aims, namely generating grant income and research papers [73]. Similarly, Madden et al. assert that current public involvement policy *“﻿creates PPI as a form of busywork in which the politics of social movements are entirely displaced by technocratic discourses of managerialism”* [30] And for Oliver and colleagues, *“far from empowering people politically, participation in research can lead to personal narratives and experiences being dominated by senior interests, leading to lack of motivation to engage again, and the ‘subjugation’ of participants”* [72]. These critical analyses of public involvement again bring us back to the question of power and the impact of how power relations play out in research settings. But such questions about impact, whilst urgent and necessary, are in the main missing from the mainstream impact agenda.

## Conclusion

This conceptual review supports our call for a more critical research agenda on public involvement in health research. We are encouraged that some groups have begun to move away from an instrumental conceptualisation of public involvement as an ‘intervention’ that has a measurable effect, and are recognising the value of continuous reflection as part of the research process [74]. We align with Staley and Barron [28], who conceptualise public involvement as a social practice of dialogue and learning between researchers and the public: an end in itself, not merely a means to an end (at worst, measured superficially as ‘bums on seats’). Critical public involvement research should explore the complexity and richness of this relationship, using methods that emphasise illumination rather than measurement [75] and asking when, why and with whom the dialogue happens or fails to happen.

We still know very little about whether and how public involvement changes power relations between researchers and the public, because this is rarely the focus of impact research. A systematic review identified some potentially useful power-focused frameworks for supporting and evaluating public involvement in research, including questions such as ‘who gets to define what empowerment is?’ and ‘whose interests are served by so-called empowerment?’ [21, 76]. And we still know very little about the wider influence of involvement on the culture of research itself, a potentially fruitful avenue for critical research.

Our findings suggest that we need more research that acknowledges, investigates and reports on the negative impacts of attempts at public involvement in health research and metrics that measure such involvement. We must ask questions about ways in which public involvement could increase inequalities, distort and suppress rather than amplify particular voices and agendas, and about how focusing on measuring impact could overshadow engagement with ethical reasons for involvement. Finally, we need to question whether the language of measurement and impact is helpful or not, and if not, what alternative language might better serve the goal of improving public involvement in health research. For example, there is a critical distinction to be drawn between ‘measuring the impact of …’ and ‘evaluating’. The former is technocratic, instrumental, largely quantitative, and summative. The latter might be reflexive, imaginative, dialogic and formative.

Much has been achieved in the field of public involvement in recent years. Much critical research remains to be done.

## Data Availability

Not applicable.

## Abbreviation

NIHR: National Institute for Health Research
